# Polymorphisms in Long Noncoding RNA-Prostate Cancer-Associated Transcript 1 Are Associated with Lung Cancer Susceptibility in a Northeastern Chinese Population

**DOI:** 10.1089/dna.2019.4834

**Published:** 2019-10-30

**Authors:** Yanhong Bi, Zhigang Cui, Hang Li, Xiaoting Lv, Juan Li, Zitai Yang, Min Gao, Ziwei Zhang, Shengli Wang, Baosen Zhou, Zhihua Yin

**Affiliations:** ^1^ Department of Epidemiology, School of Public Health, China Medical University, Shenyang, P.R. China.; ^2^ Key Laboratory of Cancer Etiology and Intervention, University of Liaoning Province, Shenyang, P.R. China.; ^3^ School of Nursing, China Medical University, Shenyang, P.R. China.

**Keywords:** lncRNA-*PCAT1*, polymorphisms, lung cancer, susceptibility, interaction

## Abstract

Long noncoding RNAs (lncRNAs) are a new class of potential biomarkers and therapeutic targets for cancer. In this study, we chose four single nucleotide polymorphisms (SNPs) in lncRNA-*PCAT1* (rs1026411 G>A, rs12543663 A>C, rs710886 T>C, and rs16901904 T>C) to investigate the association between genetic variant in lncRNA-*PCAT1* and susceptibility to lung cancer. The study was a hospital-based case–control study including 561 cancer-free controls and 468 lung cancer cases. Genotyping of four SNPs was conducted by using Taqman^®^ allelic discrimination methods. All statistical analyses were performed by using IBM SPSS Statistics 22 software. We failed to find significant associations between four SNPs and lung cancer risk in all models. However, polymorphisms in rs1026411 and rs710886 were observed to have significant associations with susceptibility to non-small cell lung cancer (AG vs. GG: odds ratio [OR]^a^ = 0.701, p* = 0.020 and AA+AG vs. GG: OR^a^ = 0.711 [superscript “a” refers to OR adjusted by age, gender, and smoking], p* = 0.017 [asterisks “*” refers to *p* adjusted by age, gender, and smoking] for rs1026411; CT vs. TT: OR^a^ = 0.723, p* = 0.047 and CC+CT vs. TT: OR^a^ = 0.729, p* = 0.038 for rs710886). Besides, the rs1026411 polymorphism had a similar association with lung adenocarcinoma risk (AG vs. GG: OR^a^ = 0.663, p* = 0.019 and AA+AG vs. GG: OR^a^ = 0.685, p* = 0.020). Polymorphisms in rs710886 and rs16901904 were observed to be associated with lung squamous cell carcinoma risk (CC+CT vs. TT: OR^a^ = 0.638, p* = 0.040 for rs710886; CC vs. TT: OR^a^ = 2.582, p* = 0.033 and CC vs. TT+CT: OR^a^ = 2.381, p* = 0.048 for rs16901904). In addition, there were no significant results in gene–environmental interactions in both additive and multiplicative models. Our results suggested that polymorphisms in lncRNA-*PCAT1* might be associated with lung cancer susceptibility in a northeastern Chinese population. The results of gene–environmental interactions were not significant in lung cancer.

## Introduction

Worldwide, lung cancer remains the leading cause of cancer morbidity and mortality for men and women, with 2.1 million new lung cancer cases (11.6% of the total cancer cases) and 1.8 million deaths (18.4% of the total cancer deaths) predicted in 2018, according to GLOBOCAN 2018 produced by the International Agency for Research on Cancer (Bray *et al.*, 2018). It is widely known that smoking is the most universal and primary risk factor for lung cancer. However, smoking cannot clarify all etiologies of lung cancer, which suggested that other environmental risk factors or genetic risk factors might play critical roles in lung carcinogenesis.

Human genome sequencing has found that protein-coding genes accounted for 3% of human DNA; however, more than 80% of our genome are actively transcribed into a miscellaneous group of RNA transcripts without potentiality for protein coding (Djebali *et al.*, 2012; ENCODE Project Consortium, 2012; Martens-Uzunova *et al.*, 2014). These transcripts were called noncoding RNAs (ncRNAs), including long noncoding RNAs (lncRNAs) and small noncoding RNAs such as microRNAs, short interfering RNAs, PIWI-interacting RNAs, and so forth.

LncRNAs are a heterogeneous group of noncoding transcripts, with more than 200 nucleotide (nt) in length, participating in a series of biological processes such as regulating chromatin dynamics, gene expression, growth, differentiation, and development (Esteller, 2011; Bhan *et al.*, 2017). Previous studies have indicated that lncRNAs are widely correlated to multiple cancers, the mutation and abnormal expression of which are closely associated with tumorigenesis, metastasis, and tumor stage (Kornfeld and Bruning, 2014; Vitiello *et al.*, 2015; Bartonicek *et al.*, 2016). Abnormal expression of lncRNAs is presented in many cancers, which can be detected in urine and/or circulating blood; therefore, lncRNAs can act as a new class of potential biomarkers and therapeutic targets for cancer, including lung cancer (Brunner *et al.*, 2012; Yan *et al.*, 2015b; Shi *et al.*, 2016; Bhan *et al.*, 2017).

Previous genome-wide association studies (GWAS) and molecular epidemiological studies have explored the underlying associations between single nucleotide polymorphisms (SNPs) in ncRNAs and disease risk. There is substantial evidence to support the association between SNPs in ncRNA and cancer risk.

Prostate cancer-associated transcript 1 (*PCAT1*) is a newly discovered lncRNA located in 8q24.21, which is first demonstrated to promote deterioration and progression of prostate cancer (Prensner *et al.*, 2011). Previous studies have shown that lncRNA-*PCAT1* plays key roles in multiple cancers, including lung cancer, by distinct mechanisms (Prensner *et al.*, 2014; Zhao *et al.*, 2015; Wen *et al.*, 2016; Bi *et al.*, 2017; Qiao *et al.*, 2017, 2018; Ren *et al.*, 2017; Xu *et al.*, 2017; Zhang *et al.*, 2017a, 2017b; Huang *et al.*, 2018; Li *et al.*, 2018). Further, lncRNA-*PCAT1* has been reported to predict a poor prognosis in many malignant tumors (Ge *et al.*, 2013; Shi *et al.*, 2015; Yan *et al.*, 2015a; Cui *et al.*, 2017; Zhang *et al.*, 2018). In addition, some researchers have conducted some studies to explore associations between genetic variation in lncRNA-*PCAT1* and cancer susceptibility in the past few years. In the study from Yuan *et al.* (2018), they selected four tagging SNPs (tagSNPs) and found that rs1902432 was a new susceptibility locus for prostate cancer in a Chinese population. A study in blabber cancer patients showed that rs710886 was an expression quantitative trait locos (eQTL) for lncRNA-*PCAT1* and may be a potential biomarker for the risk of bladder cancer (Lin *et al.*, 2017). There were also researchers who explored the association between polymorphisms on lncRNA-*PCAT1* and gastric cancer risk. They selected rs1026411 and rs12543663 in lncRNA-*PCAT1* and their results suggested that genetic variants in the two loci had no significant association with gastric cancer risk (He *et al.*, 2017).

Our previous GWAS results suggested a significant association between rs1026411 and rs12543663 polymorphisms and lung cancer risk; however, the result was based on a smaller sample size. Previous studies had indicated that rs710886 and rs16901904 were tagSNPs of lncRNA-*PCAT1* and rs710886 may be an eQTL for lncRNA-*PCAT1*. In this study, we took into account sample size and previous studies about genetic variants in lncRNA-*PCAT1* and cancer risk and chose four SNPs in lncRNA-*PCAT1* (rs1026411, rs12543663, rs710886, and rs16901904) to investigate the association between genetic variants in lncRNA-*PCAT1* and susceptibility to lung cancer. We hypothesized that these four SNPs may alter the risk of lung cancer. Therefore, we conducted a hospital-based case–control study in Shenyang, China to verify our hypothesis. Meanwhile, we also explored gene–environmental interactions.

## Materials and Methods

### Study subjects

The study was a hospital-based case–control study conducted in Shenyang city, located in northeastern China and approved by the Institutional Review Board of China Medical University. All included study subjects were unrelated Han Chinese and were divided into two groups including 468 lung cancer patients as case group and 561 cancer-free subjects as control group. Cases were chosen from several distinct hospitals in Shenyang. The inclusion criteria of the case group were as follows: (1) Cases were newly diagnosed by experienced doctors and examined by histopathological confirmation; (2) patients did not have a history of previous cancer and metastasized cancer; (3) patients had never received radiotherapy or chemotherapy; and (4) cases had ability to accept a 1.5-h interview. The control group was chosen from the physical examination centers of the hospitals just mentioned during the same period, and meanwhile, they should not have neoplasm and respiratory disease. All participants were requested to provide basic information and smoking exposure information from the moment that they were admitted to hospital and denoted 10 mL of venous blood, after signing the informed consent. The subjects who smoked more than 100 cigarettes in their lifetime were defined as smokers, and the rest were classified as nonsmokers. Besides, the control group was matched to the case group in terms of age (±5) and gender.

### SNP genotyping

The phenol-chloroform method was used to isolate DNA samples from all the study subjects' venous blood samples. We selected four SNPs in lncRNA-*PCAT1*, the minimum allele frequencies of which were greater than 0.05 in the Han Chinese population. Genotyping of the SNPs was conducted by using an Applied Biosystems 7500 FAST Real-Time PCR System (Foster City, CA) using Taqman^®^ allelic discrimination (Applied Biosystems, Foster City, CA).

### Statistical analysis

All statistical analyses were conducted by using IBM SPSS Statistics 22 software. Student's *t*-test and χ^2^ test were used to compare the differences in basic information (age, gender, and smoking status) and genotype distributions among case group and control group. The goodness-of-fit χ^2^ test was used to calculate Hardy–Weinberg's equilibrium (HWE) for SNPs in the control group. Unconditional logistic regression analysis was performed to obtain the odds ratios (ORs) and their 95% confidence intervals (CIs), which can illustrate the associations between SNPs and lung cancer risk. Crossover analysis was conducted to initially assess the interaction between four SNPs and smoking status. The further exploration of gene–environment interactions was analyzed by multiplicative interaction and the additive model. Multiplicative interaction was conducted by unconditional logistic regression, which can acquire the ORs and their 95% CIs. All ORs were adjusted by gender, age, and smoking status. The addictive interaction was evaluated through the following indicators: relative excess risk due to interaction (RERI), attributable proportion due to interaction (AP), and synergy index (S) according to the report of Andersson *et al.* (2005). All tests were two sided, and *p* < 0.05 was considered statistically significant.

## Results

### Subject characteristics

The study included a total of 1029 study subjects, whose basic information is shown in Table 1. There were 561 cancer-free subjects in the control group, the average age of which was 58.44 ± 14.55 (mean ± standard deviation [SD]). The case group was composed of 468 lung cancer patients, including 243 lung adenocarcinoma cases, 155 squamous cell carcinoma cases, and 61 small-cell lung cancer cases. The mean age of cases was 59.69 ± 10.80 (mean ± SD). The results of the Student's *t*-test and χ^2^ test for age suggested that there was no significant difference between case and control groups (*p* = 0.114 and *p* = 0.273, respectively). Similarly, there was no statistically significant differences in gender distribution between the two groups (*p* = 0.913). However, the distribution of smoking status in the two groups exhibited a significant difference and the smoking rate in the case group was significantly higher than that in the control group (*p* = 0.000). The results of HWE are shown in Supplementary Table S1. The genotype frequency distributions of these SNPs in control group were in agreement with HWE (χ^2^ = 0.185, *p* = 0.667 for rs1026411; χ^2^ = 0.187, *p* = 0.665 for rs12543663; χ^2^ = 0.202, *p* = 0.653 for rs710886; and χ^2^ = 0.237, *p* = 0.627 for rs16901904).

**Table 1.** Distribution of Demographic Variables in Lung Cancer Case and Control Groups

	*Case (%) (*n* = 468)*	*Control (%) (*n* = 561)*	p
Age (mean ± SD)	59.69 ± 10.80	58.44 ± 14.55	0.114
Age			
≤59	217 (46.4)	241 (43.0)	0.273
>59	251 (53.6)	320 (57.0)	
Gender			
Male	242 (51.7)	292 (52.0)	0.913
Female	226 (48.3)	269 (48.0)	
Smoking exposure			
Never	231 (49.4)	425 (75.8)	0.000
Ever	237 (50.6)	136 (24.2)	
Histology			
Non-small cell lung cancer	407 (87.0)		
Lung adenocarcinoma	243 (51.9)		
Squamous cell carcinoma	155 (33.1)		
Small cell lung cancer	61 (13.0)		
Other	9 (1.9)		

SD, standard deviation.

### Genotype distribution of SNPs and their associations with lung cancer susceptibility

The associations of polymorphisms in lncRNA-*PCAT1* with susceptibility to lung cancer and non-small cell lung cancer are shown in Table 2. No statistically significant associations were found between four SNPs and lung cancer risk in all models. However, we found that genetic variants in rs1026411 and rs710886 had significant associations with susceptibility to non-small cell lung cancer (AG vs. GG: OR^a^ = 0.701, 95% CI = 0.520–0.946, p* = 0.020 and AA+AG vs. GG: OR^a^ = 0.711, 95% CI = 0.538–0.940, p* = 0.017 for rs1026411; CT vs. TT: OR^a^ = 0.723, 95% CI = 0.525–0.995, p* = 0.047 and CC+CT vs. TT: OR^a^ = 0.729, 95% CI = 0.541–0.982, p* = 0.038 for rs710886). In addition, we failed to find a significant association between polymorphisms in rs12543663 and rs16901904 and the risk of lung cancer and non-small cell lung cancer. Table 3 described the results of associations between polymorphisms in lncRNA-*PCAT1* and susceptibility to the subtypes of lung cancer, including lung adenocarcinoma and squamous cell carcinoma. We found that polymorphisms in rs1026411 had a significant association with lung adenocarcinoma risk (AG vs. GG: OR^a^ = 0.663, 95% CI = 0.470–0.936, p* = 0.019 and AA+AG vs. GG: OR^a^ = 0.685, 95% CI = 0.497–0.943, p* = 0.020). In squamous cell carcinoma, CC or CT genotype of rs710886 had a significantly decreased risk compared with TT genotype carrier (OR^a^ = 0.638, 95% CI = 0.416–0.980, p* = 0.040). Besides, polymorphisms in rs16901904 also showed significant results in this subgroup. In comparison with the TT genotype carrier, the risk of squamous cell carcinoma significantly increased in the CC genotype (OR^a^ = 2.582, 95% CI = 1.078–6.186, p* = 0.033); meanwhile, the recessive model of rs16901904 also presented significant results (OR^a^ = 2.381, 95% CI = 1.009–5.620, p* = 0.048).

**Table 2.** Association Between Polymorphisms in the Four Single Nucleotide Polymorphisms and Risk of Lung Cancer and Nonsmall Cell Lung Cancer

			*Lung cancer*			*Non-small cell lung cancer*		
*SNP*	*Genotype*	*Controls (%) (*n* = 561)*	*Case (%) (*n* = 468)*	*OR*^a^*(95% CI)*	p^*^	*Case (%) (*n* = 407)*	*OR*^a^*(95% CI)*	p^*^
rs1026411	GG	196 (34.9)	184 (39.3)	1.00 (Ref)		168 (41.3)	1.00 (Ref)	
	AG	267 (47.6)	204 (43.6)	0.761 (0.569–1.018)	0.066	171 (42.0)	0.701 (0.520–0.946)	0.020
	AA	98 (17.5)	80 (17.1)	0.776 (0.531–1.136)	0.192	68 (16.7)	0.738 (0.498–1.093)	0.130
	AA+AG vs. GG			0.765 (0.583–1.005)	0.054		0.711 (0.538–0.940)	0.017
	AA vs. GG+AG			0.904 (0.640–1.275)	0.564		0.895 (0.626–1.280)	0.544
	G allele	659 (58.7)	572 (61.1)	1.00 (Ref)		507 (62.3)	1.00 (Ref)	
	A allele	463 (41.3)	364 (38.9)	0.906 (0.759–1.081)	0.273	307 (37.7)	0.862 (0.716–1.037)	0.115
rs12543663	AA	472 (84.1)	394 (84.2)	1.00 (Ref)		342 (84.0)	1.00 (Ref)	
	AC	86 (15.3)	70 (15.0)	1.039 (0.720–1.498)	0.838	61 (15.0)	1.033 (0.708–1.509)	0.865
	CC	3 (0.5)	4 (0.9)	1.369 (0.267–7.010)	0.707	4 (1.0)	1.546 (0.305–7.840)	0.599
	AC+CC vs. AA			1.051 (0.734–1.505)	0.787		1.052 (0.726–1.525)	0.788
	CC vs. AA+AC			1.361 (0.266–6.962)	0.712		1.538 (0.304–7.792)	0.603
	A	1030 (91.8)	858 (91.7)	1.00 (Ref)		745 (91.5)	1.00 (Ref)	
	C	92 (8.2)	78 (8.3)	1.018 (0.743–1.395)	0.913	69 (8.5)	1.037 (0.748–1.437)	0.828
rs710886	TT	150 (26.7)	140 (29.9)	1.00 (Ref)		129 (31.7)	1.00 (Ref)	
	CT	275 (49.0)	216 (46.2)	0.785 (0.574–1.072)	0.127	184 (45.2)	0.723 (0.525–0.995)	0.047
	CC	136 (24.2)	112 (23.9)	0.801 (0.557–1.153)	0.232	94 (23.1)	0.740 (0.509–1.077)	0.116
	CC+CT vs. TT			0.790 (0.590–1.058)	0.114		0.729 (0.541–0.982)	0.038
	CC vs. TT+CT			0.934 (0.688–1.268)	0.662		0.906 (0.659–1.245)	0.542
	T	575 (51.2)	496 (53.0)	1.00 (Ref)		442 (54.3)	1.00 (Ref)	
	C	547 (48.8)	440 (47.0)	0.933 (0.784–1.109)	0.430	372 (45.7)	0.885 (0.738–1.060)	0.184
rs16901904	TT	364 (64.9)	294 (62.8)	1.00 (Ref)		251 (61.7)	1.00 (Ref)	
	CT	178 (31.7)	151 (32.3)	1.124 (0.847–1.493)	0.418	134 (32.9)	1.155 (0.862–1.547)	0.336
	CC	19 (3.4)	23 (4.9)	1.515 (0.772–2.972)	0.227	22 (5.4)	1.705 (0.866–3.358)	0.123
	CC+CT vs. TT			1.163 (0.885–1.527)	0.279		1.208 (0.913–1.600)	0.186
	CC vs. TT+CT			1.457 (0.748–2.839)	0.268		1.625 (0.831–3.176)	0.156
	T	906 (80.7)	739 (79.0)	1.00 (Ref)		636 (78.1)	1.00 (Ref)	
	C	216 (19.3)	197 (21.0)	1.118 (0.901–1.388)	0.311	178 (21.9)	1.174 (0.939–1.467)	0.158

^a^ OR adjusted by age, gender, and smoking.

^*^ *p* adjusted by age, gender, and smoking.

CI, confidence interval; OR, odds ratio; Ref, reference.

**Table 3.** Association Between Polymorphisms in the Four Single Nucleotide Polymorphisms and Risk of Lung Adenocarcinoma and Squamous Cell Lung Cancer

			*Lung adenocarcinoma*			*Squamous cell carcinoma*		
*SNP*	*Genotype*	*Controls (%) (*n* = 561)*	*Case (%) (*n* = 243)*	*OR*^a^*(95% CI)*	p^*^	*Case (%) (*n* = 155)*	*OR*^a^*(95% CI)*	p^*^
rs1026411	GG	196 (34.9)	104 (42.8)	1.00 (Ref)		62 (40.0)	1.00 (Ref)	
	AG	267 (47.6)	98 (40.3)	0.663 (0.470–0.936)	0.019	67 (43.2)	0.678 (0.437–1.051)	0.082
	AA	98 (17.5)	41 (16.9)	0.744 (0.474–1.166)	0.197	26 (16.8)	0.698 (0.392–1.243)	0.222
	AA+AG vs. GG			0.685 (0.497–0.943)	0.020		0.684 (0.454–1.029)	0.069
	AA vs. GG+AG			0.927 (0.614–1.401)	0.720		0.867 (0.513–1.465)	0.594
	G allele	659 (58.7)	306 (63.0)	1.00 (Ref)		191 (61.6)	1.00 (Ref)	
	A allele	463 (41.3)	180 (37.0)	0.837 (0.672–1.042)	0.112	119 (38.4)	0.887 (0.685–1.148)	0.361
rs12543663	AA	472 (84.1)	203 (83.5)	1.00 (Ref)		133 (85.8)	1.00 (Ref)	
	AC	86 (15.3)	38 (15.6)	1.062 (0.689–1.635)	0.786	20 (12.9)	0.881 (0.494–1.572)	0.669
	CC	3 (0.5)	2 (0.8)	1.146 (0.160–8.205)	0.892	2 (1.3)	2.640 (0.351–19.858)	0.346
	AC+CC vs. AA			1.065 (0.697–1.628)	0.772		0.942 (0.539–1.646)	0.833
	CC vs. AA+AC			1.136 (0.159–8.120)	0.899		2.688 (0.358–20.202)	0.337
	A	1030 (91.8)	444 (91.4)	1.00 (Ref)		286 (92.3)	1.00 (Ref)	
	C	92 (8.2)	42 (8.6)	1.059 (0.723–1.551)	0.768	24 (7.7)	0.939 (0.588–1.500)	0.794
rs710886	TT	150 (26.7)	75 (30.9)	1.00 (Ref)		52 (33.5)	1.00 (Ref)	
	CT	275 (49.0)	111 (45.7)	0.744 (0.514–1.076)	0.116	68 (43.9)	0.645 (0.407–1.023)	0.063
	CC	136 (24.2)	57 (23.5)	0.787 (0.511–1.212)	0.277	35 (22.6)	0.624 (0.362–1.077)	0.090
	CC+CT vs. TT			0.758 (0.537–1.070)	0.115		0.638 (0.416–0.980)	0.040
	CC vs. TT+CT			0.948 (0.658–1.367)	0.775		0.817 (0.511–1.307)	0.399
	T	575 (51.2)	261 (53.7)	1.00 (Ref)		172 (55.5)	1.00 (Ref)	
	C	547 (48.8)	225 (46.3)	0.906 (0.732–1.122)	0.365	138 (44.5)	0.843 (0.655–1.086)	0.186
rs16901904	TT	364 (64.9)	153 (63.0)	1.00 (Ref)		93 (60.0)	1.00 (Ref)	
	CT	178 (31.7)	81 (33.3)	1.106 (0.791–1.548)	0.555	50 (32.3)	1.268 (0.824–1.951)	0.281
	CC	19 (3.4)	9 (3.7)	1.214 (0.515–2.862)	0.657	12 (7.7)	2.582 (1.078–6.186)	0.033
	CC+CT vs. TT			1.116 (0.807–1.544)	0.507		1.400 (0.931–2.106)	0.106
	CC vs. TT+CT			1.174 (0.502–2.746)	0.711		2.381 (1.009–5.620)	0.048
	T	906 (80.7)	387 (79.6)	1.00 (Ref)		236 (76.1)	1.00 (Ref)	
	C	216 (19.3)	99 (20.4)	1.073 (0.822–1.400)	0.604	74 (23.9)	1.315 (0.974–1.776)	0.073

^a^ OR adjusted by age, gender, and smoking.

^*^ *p* adjusted by age, gender, and smoking.

### Interaction between four SNPs and smoking status

Table 4 presented the crossover analysis results of four SNPs and smoking status to investigate the gene–environmental interaction. We selected non-smokers with a protective genotype of four SNPs (AA+AG genotype of rs1026411, AA genotype of rs12543663, CC+CT genotype of rs710886, and TT genotype of rs16901904) as the reference group, respectively. The results of the crossover analysis showed that smokers with both protective and dangerous genotypes significantly increased the risk of lung cancer and nonsmall cell lung cancer compared with nonsmokers with protective genotypes. These results indicated that there might be gene–environmental interactions, for the reason that we used the additive model and the multiplicative model to further investigate the gene–environmental interaction; the results are presented in Table 5 and 6. We did not find significant results of the gene–environmental interaction on both the additive and multiplicative scales.

**Table 4.** Crossover Analysis of Interaction Between Risk Genotypes of Four Single Nucleotide Polymorphisms and Smoking Exposure in Lung Cancer and Nonsmall Cell Lung Cancer

				*Lung cancer*			*Non-small cell lung cancer*		
*SNPs*	*Genotype*	*Smoking exposure*	*Controls (%) (*n* = 561)*	*Case (%) (*n* = 468)*	*OR*^a^*(95% CI)*	p^*^	*Cases (%) (*n* = 407)*	*OR*^a^*(95% CI)*	p^*^
rs1026411	AA+AG	Never	272 (48.5)	137 (29.3)	1.00 (Ref)		122 (30.0)	1.00 (Ref)	
	GG	Never	153 (27.3)	94 (20.1)	1.300 (0.924–1.828)	0.132	89 (21.9)	1.369 (0.965–1.942)	0.079
	AA+AG	Ever	93 (16.6)	147 (31.4)	7.783 (4.949–12.238)	0.000	117 (28.7)	6.731 (4.219–10.740)	0.000
	GG	Ever	43 (7.7)	90 (19.2)	10.258 (6.103–17.240)	0.000	79 (19.4)	9.935 (5.830–16.929)	0.000
rs12543663	AA	Never	353 (62.9)	191 (40.8)	1.00 (Ref)		175 (43.0)	1.00 (Ref)	
	CC+AC	Never	72 (12.8)	40 (8.5)	1.024 (0.661–1.588)	0.915	36 (8.8)	1.006 (0.639–1.583)	0.979
	AA	Ever	119 (21.2)	203 (43.4)	7.586 (5.017–11.471)	0.000	167 (41.0)	6.626 (4.337–10.123)	0.000
	CC+AC	Ever	17 (3.0)	34 (7.3)	8.406 (4.262–16.577)	0.000	29 (7.1)	7.649 (3.803–15.387)	0.000
rs710886	CC+CT	Never	310 (55.3)	161 (34.4)	1.00 (Ref)		145 (35.6)	1.00 (Ref)	
	TT	Never	115 (20.5)	70 (15.0)	1.290 (0.894–1.862)	0.174	66 (16.2)	1.343 (0.923–1.956)	0.123
	CC+CT	Ever	101 (18.0)	167 (35.7)	7.897 (5.099–12.232)	0.000	133 (32.7)	6.754 (4.306–10.596)	0.000
	TT	Ever	35 (6.2)	70 (15.0)	9.677 (5.608–16.698)	0.000	63 (15.5)	9.618 (5.502–16.813)	0.000
rs16901904	TT	Never	270 (48.1)	144 (30.8)	1.00 (Ref)		130 (31.9)	1.00 (Ref)	
	CC+CT	Never	155 (27.6)	87 (18.6)	1.085 (0.770–1.530)	0.640	81 (19.9)	1.104 (0.776–1.570)	0.584
	TT	Ever	94 (16.8)	150 (32.1)	7.221 (4.619–11.287)	0.000	121 (29.7)	6.218 (3.924–9.853)	0.000
	CC+CT	Ever	42 (7.5)	87 (18.6)	9.467 (5.634–15.906)	0.000	75 (18.4)	8.827 (5.185–15.029)	0.000

^a^ OR adjusted by age and gender.

^*^ *p* adjusted by age and gender.

**Table 5.** Addictive Interaction Between Four Single Nucleotide Polymorphisms in Prostate Cancer-Associated Transcript 1 and Smoking Exposure in Lung Cancer and Nonsmall Cell Lung Cancer

		*Lung cancer*		*Nonsmall cell lung cancer*	
*SNPs*	*Measure*	*Estimate*	*95% CI*	*Estimate*	*95% CI*
rs1026411	RERI	0.797	−0.947 to 2.542	0.994	−0.741 to 2.730
	AP	0.192	−0.173 to 0.556	0.243	−0.109 to 0.594
	S	1.338	0.724 to 2.475	1.473	0.772 to 2.811
rs12543663	RERI	0.517	−1.786 to 2.820	0.602	−1.602 to 2.805
	AP	0.140	−0.410 to 0.689	0.175	−0.371 to 0.721
	S	1.237	0.510 to 3.003	1.327	0.512 to 3.439
rs710886	RERI	0.495	−1.291 to 2.281	0.806	−0.982 to 2.594
	AP	0.129	−0.293 to 0.550	0.209	−0.184 to 0.602
	S	1.210	0.622 to 2.354	1.395	0.694 to 2.803
rs16901904	RERI	0.839	−0.809 to 2.488	0.950	−0.652 to 2.552
	AP	0.216	−0.145 to 0.577	0.256	−0.099 to 0.611
	S	1.411	0.736 to 2.703	1.540	0.766 to 3.098

AP, attributable proportion due to interaction; RERI, relative excess risk due to interaction; S, synergy index.

**Table 6.** Multiplicative Interaction Between Four Single Nucleotide Polymorphisms in Prostate Cancer-Associated Transcript 1 and Smoking Exposure in Lung Cancer and Nonsmall Cell Lung Cancer

	*Lung cancer*		*Nonsmall cell lung cancer*	
*SNP*	*OR*^a^	p^*^	*OR*^a^	p^*^
rs1026411 (GG vs. AA+AG)	1.300 (0.924–1.828)	0.132	1.369 (0.965–1.942)	0.079
Smoking exposure	7.783 (4.949–12.238)	0.000	6.731 (4.219–10.740)	0.000
Interaction	1.014 (0.576–1.786)	0.962	1.078 (0.602–1.933)	0.800
rs12543663 (CC+AC vs. AA)	1.024 (0.661–1.588)	0.915	1.006 (0.639–1.583)	0.979
Smoking exposure	7.586 (5.017–11.471)	0.000	6.626 (4.337–10.123)	0.000
Interaction	1.082 (0.501–2.337)	0.842	1.147 (0.518–2.541)	0.735
rs710886 (TT vs. CC+CT)	1.290 (0.894–1.862)	0.174	1.343 (0.923–1.956)	0.123
Smoking exposure	7.897 (5.099–12.232)	0.000	6.754 (4.306–10.596)	0.000
Interaction	0.950 (0.519–1.738)	0.868	1.060 (0.570–1.970)	0.854
rs16901904 (CC+CT vs. TT)	1.085 (0.770–1.530)	0.640	1.104 (0.776–1.570)	0.584
Smoking exposure	7.221 (4.619–11.287)	0.000	6.218 (3.924–9.853)	0.000
Interaction	1.208 (0.682–2.138)	0.517	1.286 (0.713–2.322)	0.403

^a^ OR adjusted by age and gender.

^*^ *p* adjusted by age and gender.

## Discussion

Lung cancer is an extremely complicated malignant tumor with multiple etiologies. As we all know, smoke is not able to explain the full causes of lung cancer. Treating lung cancer patients better and more effectively is critical to identify other early diagnostic biomarkers. LncRNAs are usually classified as follows: sense, antisense, bidirectional, intronic, and intergenic (Xu *et al.*, 2014). Through transcriptome sequencing and microarrays analysis, multiple lncRNAs are found to be associated with susceptibility to lung cancer and its subtype (Wang *et al.*, 2014; White *et al.*, 2014; Xu *et al.*, 2014).

LncRNA-*PCAT1* was first identified as a biomarker for prostate cancer by transcriptome sequencing and it was also named accordingly (Prensner *et al.*, 2011). Previous studies have shown that lncRNA-*PCAT1* plays key roles in many diseases by distinct mechanisms. A study from Zhang *et al.* (2017b) showed that lncRNA-*PCAT1* affects extrahepatic cholangiocarcinoma (ECC) progression by the Wnt/β-catenin-signaling pathway and *PCAT1* may be a potential therapeutic target for ECC treatment. Another study showed that lncRNA-*PCAT1* contributes to prostate cancer risk by regulating FSCN1 via miR-145-5p (Xu *et al.*, 2017). Zhao *et al.* (2015) showed that lncRNA-*PCAT1* was correlated with cell proliferation, migration, and invasion of nonsmall cell lung cancer cells, which suggested a novel therapeutic target of lung cancer. The study from Li *et al.* (2018) suggested that lncRNA-*PCAT1* might influence the development of nonsmall cell lung cancer via the miR-149-59/LRIG2 axis.

Previous studies showed that there were a majority of GWAS or trait-associated loci in the 8q24 gene desert region, a well-known genetic region (Huppi *et al.*, 2012; Panagiotou *et al.*, 2015). LncRNA-*PCAT1* is located at chromosome 8q24.21. Yuan *et al.* (2018) showed that rs1902432 polymorphisms in lncRNA-*PCAT1* had a significant association with prostate cancer risk in the additive model, co-dominant model, and recessive model. A study from Lin *et al.* (2017) suggested that rs710886 (A>G), an eQTL for lncRNA-*PCAT1*, significantly reduced bladder cancer risk (OR = 0.86, *p* = 0.046).

This case–control study recruited 468 lung cancer cases and 561 cancer-free controls, which were matched in age and gender between the case and control groups. Through this research, we found that polymorphisms in lncRNA-*PCAT1* had a significant association with lung cancer risk. Rs1902432 was in high linkage disequilibrium (*r*^2^ = 0.99) with rs1026411, based on HaploReg v4.1 (Ward and Kellis, 2012). In our study, genetic variants in rs1026411 may be protective factors in non-small cell lung cancer, which is different from rs1902432 in prostate cancer. The possible reason is that pathogenic mechanisms were different in distinct cancers. The analysis results of HaploReg database (Ward and Kellis, 2012) also showed that rs710886 is an eQTL for *PCAT1* and is located at a region that overlaps with enhancer histone marks in two tissues. Our results indicated that rs710886 polymorphisms were significantly associated with reduced risk of non-small cell lung cancer. The results were consistent with those in bladder cancer. Besides, we also found some statistically significant association in the subtype of lung adenocarcinoma and squamous cell carcinoma. Although rs16901904 polymorphisms had no statistically significant association with the risk of lung cancer, non-small cell lung cancer, and lung adenocarcinoma, we find that rs16901904 polymorphisms were associated with the risk of lung squamous cell carcinoma. The reason might be that the result was a false positive, which was caused by a smaller sample size after stratification.

All study subjects included in this study were based on a set of strict inclusion and exclusion criteria. By selecting newly diagnosed patients to the case group, this study effectively avoids Neyman bias, which frequently occurs in case–control studies. The control group was matched to the case group in age and gender. All unconditional logistic regression analyses were adjusted by age, gender, and smoking status; by this means, confounding bias can be reduced effectively. However, there were still some limitations in this study. First, there may be Berkson's bias in this study, since all study subjects were selected from the hospital. Second, all controls were selected from the medical examination center of hospitals, which could not represent the whole control population to some extent. Third, there was a lack of further functional studies to confirm our results.

## Conclusion

Genetic variants in lncRNA-*PCAT1* may be associated with lung cancer susceptibility in a northeastern Chinese population. The interaction between lncRNA-*PCAT1* and smoking status does not exist in this study.

## Supplementary Material

Supplemental data
