# Supplementary material for: Polymorphisms in Long Noncoding RNA-Prostate Cancer-Associated Transcript 1 Are Associated with Lung Cancer Susceptibility in a Northeastern Chinese Population
Source: DNA Cell Biol. 2019 Oct 30;38(11):1357–65. doi: 10.1089/dna.2019.4834 (PMC6822575; doi:10.1089/dna.2019.4834)
Supplement: Supplemental data [file Supp_Table1.pdf]

Supplementary Data

Supplementary Table S1. The Hardy–Weinberg Equilibrium for These Single Nucleotide Polymorphisms in Control Group

SNP	χ^2	p
rs1026411	0.185	0.667
rs12543663	0.187	0.665
rs710886	0.202	0.653
rs16901904	0.237	0.627

SNP, single nucleotide polymorphism.
